# Pharmacological management in orofacial pain: a retrospective, observational study of treatment decisions and contributing factors

**DOI:** 10.22514/jofph.2026.009

**Published:** 2026-01-12

**Authors:** Diya Mundackal, Aleksandra Zumbrunn Wojczyńska, Mutlu Özcan, Nenad Lukic, Vera Colombo

**Affiliations:** ^1^Clinic of Masticatory Disorders and Dental Biomaterials, Center for Dental Medicine, University of Zurich, 8032 Zurich, Switzerland

**Keywords:** Clinical study, Orofacial pain, Pharmacological therapy, Pharmacotherapy, Overtreatment, Undertreatment, Temporomandibular disorders

## Abstract

**Background**: The study evaluated how often pharmacological therapies were started, modified, or discontinued after a consultation in a sample of orofacial pain patients and identified potential factors associated with treatment choices in the pharmacological management of orofacial pain. **Methods**: For this study, patient files (N = 208) originating from the daily routine of the Orofacial Pain Unit, University of Zurich (January 2017–December 2022) were analysed. Demographics, lifestyle, pain characteristics, diagnosis, and pharmacological therapy pre- and post- consultation with an orofacial pain specialist were recorded. Changes in pharmacotherapy, pain perception, and therapeutic success were assessed. Descriptive statistics, paired McNemar and chi-square tests were conducted. **Results**: A total of 208 patients were included in the study (64.4% females, mean age 45.9 years). The mean pain intensity was 6.93 for maximum pain and 4.62 for average pain. The most common pain locations were the face (64.3%), followed by the head (33.3%). At the initial consultation, 51.4% of patients were already using pharmacological therapy. The most common pre-diagnosis medications were non-steroidal anti-inflammatory drugs (NSAIDs) (44.9%), antidepressants with pain-modulating properties (9.3%), and magnesium (7.5%). After consultation, myofascial orofacial pain was the most common diagnosis (50.5%). The prescription of medications increased significantly to 74.5% (*p * < 0.001). Topical NSAIDs (64.0%) and magnesium supplements (40.0%) were the most prescribed. A significant relationship between therapy changes and diagnosis was observed, particularly for myofascial pain (*p* = 0.024) and temporomandibular joint disorders (*p * < 0.001). Therapy outcomes were positive for 67.0% of the observed patients. **Conclusions**: Age, psychological distress, and pain location significantly influenced pharmacological management of orofacial pain. Pharmacological therapy differed between before and after consultation at the Orofacial Pain Unit. Accurate diagnosis and a multidisciplinary approach to treatment can significantly improve therapy success.

## 1. Introduction

Pain, a common human experience, is a complex multifaceted phenomenon that affects millions of individuals worldwide. It is more than just a physical sensation but an intricate interplay of biological, psychological and social components [1]. Chronic pain is a major healthcare challenge, frequently resulting in increased suffering, reduced quality of life and high medical expenses. Studies indicate that around 30% of the global population use some pain medication [2]. Among the various types of chronic pain, orofacial pain represents a unique condition due to its vast array of underlying causes, including dental, myofascial, articular, and neuropathic issues, as well as contributing psychological factors.

Orofacial pain is perceived in many regions of the face and oral cavity, divided into macro areas [3, 4, 5]. It includes various acute and chronic pain conditions involving structures supplied by the trigeminal nerve, including the teeth, oral tissues, jaw muscles, and the temporomandibular joint (TMJ) [6]. Studies have shown that approximately 10% of the adult population suffer from chronic orofacial pain [7]. Acute orofacial pain is most often related to teeth [8], whilst chronic orofacial pain is most often related to temporomandibular disorders (TMD) [9]. Studies suggest that incidences of orofacial pain are rising and are more prevalent in women [10]. Additionally, in younger individuals, myofascial pain due to psychosocial stress is more common, whereas in middle-aged individuals, there is a shift to chronic pain from TMJ related osteoarthritis [11].

Orofacial pain manifests by a wide range of symptoms, emphasizing the complexity of the diagnostic process. Symptoms may include pain described as throbbing, sharp, or diffuse, and either localized or radiating to neighbouring regions [12]. Furthermore, patients with other chronic health conditions may experience a heightened level of orofacial pain considering the overlapping characteristics of pain syndromes [13]. On the other hand, overlapping symptoms of various conditions and the presence of neurological pathologies can lead to misdiagnosis and subsequently an inaccurate treatment [12, 14].

The management of orofacial pain often requires a combination of pharmacological and non-pharmacological ways to tackle the underlying cause of pain [15]. Recent guidelines suggest that TMD therapy should primarily focus on cognitive-behavioural and supported self-management recommendations, whereas pharmacotherapy is regarded as a second-line therapeutic option, to be employed alongside the interim use of oral appliances, and leave surgical interventions restricted to very selected cases [16]. These approaches have been shown to increase functionality, reduce pain and hence improve the overall quality of life. Still, pharmacological interventions continue to be extensively utilized in clinical practice [17]. The therapeutic effect of pharmacotherapy in TMD is attributable to neuromodulation, defined as the process by which the release or action of excitatory neurotransmitters is diminished, or alternatively, by which the properties of nociceptive neurons or their pathways are modified [16].

The pharmacological treatment in orofacial pain patients includes the use of a broad range of medication. The most common pharmacological approach includes the intake of analgesics, anti-inflammatory drugs, and muscle relaxants, among others [17]. A retrospective study by Sotorra-Figuerola *et al.* [18], has highlighted that Nonsteroidal Anti-inflammatory Drugs (NSAID) are widely used due to their proven effectiveness in reducing inflammation especially in TMD and dental pain. However, systemic pharmacotherapy is associated with a risk of systemic adverse effects, including gastrointestinal complications or medication overuse headache. Studies have shown that limitations in daily activities are strong predictors in the usage of pharmacotherapy, while age, gender and education may also play a role.

Nevertheless, the risk of overtreatment and undertreatment poses a concern in clinical practices. According to the U.S. National Library of Medicine (NLM), “Overtreatment” is defined as “Remedial treatment or preventive procedures of a disease which is done too frequently or excessively often from overdiagnosis” [19], whereas “Undertreatment” indicates a “Remedial treatment or preventive procedures of a disease which is done too infrequently or not enough to control disease diagnosis or treatment” [20]. Overtreatment can lead to unnecessary side effects and an increased treatment cost, whereas undertreatment can lead to pain persistence and chronification resulting in a reduced quality of life. A qualitative study showed that patients with chronic orofacial pain often feel their pain is treated insufficiently, which further emphasizes the need for a deeper understanding for both treatment effectiveness as well as better patient experiences [21].

The aim of this study is to provide insight into trends in pharmacological pain management among patients with orofacial pain and to identify the factors influencing medication use and therapeutic decision-making. Specifically, the study evaluates the prevalence of pharmacotherapy in a sample of patients with orofacial pain, examines the frequency with which pre-existing pharmacological regimens were recommended or modified following specialist consultation, and analyses the factors associated with pharmacological prescription.

## 2. Materials and methods

### 2.1 Subjects

Data analysed in this study originates from a pool of patients with possible orofacial pain or non-painful masticatory disorders who were referred to the Orofacial Pain Unit of the Center for Dental Medicine in Zurich between 2017 and 2022. Given the extensive number of patients’ records available at the clinic, a sample of two hundred and eight patients was collected. In order to minimize selection bias, forty files per year were randomly selected for data extraction ensuring a representative dataset distributed throughout the selected period. Inclusion criteria required that they were patients with orofacial pain that signed a written consent for further use of their data. For patients under eighteen years of age a written consent for further use of the child’s data was required to be signed by the custodian. Patients who did not provide written consent were excluded from the study. The Ethics Committee of the state of Zurich approved the study protocol (KEK Nr. 2023-01966).

### 2.2 Study design

This was a retrospective, descriptive study that was conducted to evaluate how often a therapy was changed after an appointment at the Orofacial Pain Unit, Clinic of Masticatory Disorders and Dental Biomaterials, Center for Dental Medicine, University of Zurich. The study included randomly selected patients with orofacial pain who visited the clinic between 2017 and 2022 and had signed a written informed consent for further use of their health-related data. The primary variable of interest was the percentage of therapy change, where medication was discontinued or added after a consultation with an orofacial pain specialist. The study complied with the regulatory requirements of the Human Research Act (HRA) and the Human Research with the Exception of Clinical trials (HRO).

### 2.3 Study procedure

Upon selection of patient files, data extraction was manually performed by the research team and entered into an Excel spreadsheet specifically designed for this study. General demographic information, including age, gender, and maximum employment load, was extracted to define the characteristics of the patient population attending the clinic for their initial visit. Lifestyle factors, such as smoking, alcohol consumption, and drug use, were recorded alongside medical history variables, including the presence or absence of comorbidities, mental distress, and pre-existing diagnoses.

The presence of mental distress was based on self-assessment of patients during a screening evaluation with online questionnaires. The following pain-related characteristics were tabulated: maximum and average pain intensity (Numeric Rating Scale 0–10), pain duration (<3 months; 3–6 months; 6 months–2 years; 2–5 years; >5 years), and pain location (face, head, arm). Additionally, prior pharmacological treatments were documented as patient self-report. All data were collected as primary information at the initial visit, prior to the assignment of a diagnosis or treatment plan by an orofacial pain specialist.

Diagnoses formulated based on the clinical examination and medical history at the first or later visits of the Orofacial Pain Unit were grouped into categories, based on the International Classification of Orofacial Pain (ICOP) [22] as following:

1. Orofacial pain or non-painful disorders attributed to dentoalveolar and related structures.

2. Myofascial orofacial pain.

3. Temporomandibular joint (TMJ) pain and non-painful TMJ disorders.

4. Orofacial pain attributed to cranial nerve lesions or disease.

5. Orofacial pain resembling primary headache presentations.

6. Idiopathic orofacial pain.

7. Others (occlusal hypervigilance, obstructive sleep apnoea syndrome, tinnitus).

Based on the diagnosis, therapies were prescribed to the patients. These included both pharmacological and non-pharmacological treatments. Medications prescribed prior to and after the visit were divided into subgroups based on their type and therapeutic purpose. The categories were:

1. NSAIDs (topical and/or systemic).

2. Magnesium supplements.

3. Pain-modulating antidepressants (including amitriptyline/ketamine gel).

4. Vitamin B supplements.

5. Antiseptic mouth rinses.

6. Antidepressants without pain modulation.

7. Antiepileptic drugs.

8. Topical agents for dentin hypersensitivity.

9. Steroids.

10. Proton pump inhibitors (PPI).

11. Non-NSAIDs (Opioid analgesics, non-opioid analgesics/anti-inflammatory).

12. Triptans.

13. Muscle relaxants.

14. Benzodiazepines.

15. Atypical antipsychotics.

16. Topical anaesthetic creams.

17. Capsaicin mouth rinses.

For patients with prior medication, therapy changes were categorized as continuation, modification or discontinuation.

Pain perception was recorded using the Visual Analog Scale (VAS) at the initial visit. Therapeutic success was defined as a ≥30% reduction in pain intensity after three months of conservative treatment and an average pain intensity ≤50 mm on the VAS during the last month of follow-up [23]. Furthermore, the subjective change in pain level at the last visit in comparison to the first visit, categorized as improvement, worsening, or no change, was used to analyse the association between the use of pharmacological therapy and overall pain perception.

### 2.4 Data analysis and statistics

A descriptive and interferential-statistical analysis was conducted using statistical software, IBM SPSS Statistics Software (SPSS Version 29, IBM Corporation, Armonk, NY, USA).

Demographics, lifestyle, and clinical characteristics of the study population at the initial visit (baseline) were assessed with descriptive statistics. Continuous variables were described as mean and standard deviation (SD). Categorical variables were summarized as frequencies and percentages.

The distribution of diagnoses and the frequency of pharmacological treatments before and after consultation with the orofacial pain specialist were analysed. Chi-square tests were used to evaluate the association between patient characteristics at baseline and the use of pharmacological therapy prior to the Orofacial Pain Consultation. Changes in the diagnoses and pharmacological therapies globally and pro-diagnosis before/after consultation were assessed using the non-parametric McNemar Change test for related samples.

The frequency of therapy success was evaluated globally and across diagnoses. Furthermore, the association between the presence of pharmacological therapy and therapeutic success was determined with Chi-square statistics.

## 3. Results

### 3.1 Patient characteristics at baseline

A total of 208 patients were included in the study, predominantly consisting of females (64.4%). The mean age of the study population was 45.9 (SD = 17.7) years, ranging from 12 years to 91 years. The most represented age group was 40–60 years old (39.9%). The mean maximum pain intensity was 6.93 (SD = 2.5) and the mean average pain intensity was 4.62 (SD = 2.5). Pain duration ranged from less than 3 months to more than 5 years with a mean of 40.2 (SD = 62.7) months. Pain was primarily localized to the face (64.3%), followed by the head (33.3%) and the arm (2.4%) (Table 1). Systemic diseases were reported by 61.2% of the patients.

**Table 1.** Characteristics of the patient population at the time of first consultation.

Group		Frequency (%)
Gender		
	Male	35.6
	Female	64.4
Age group at first consultation (yr)		
	Age ≤20	8.7
	20 < age ≤ 40	31.7
	40 < age ≤ 60	39.9
	60 < age ≤ 80	15.4
	Age >80	4.3
Location of pain		
	Face	64.3
	Head	33.3
	Arm	2.4
Timeframe of pain		
	<3 mon	16.8
	3–6 mon	10.2
	6 mon–2 yr	33.5
	2–5 yr	17.3
	>5 yr	22.3

History of head and neck injuries was reported in 32.2% of the patients, whereas 21.1% reported mental distress (Table 2). Lifestyle characteristics at baseline included alcohol consumption in 47.6% of patients, smoking in 19.3%, and recreational drug use in 2.4%.

**Table 2.** Prevalence of comorbidities and lifestyle situation in the patient population at the time of the first consultation.

Comorbidity/lifestyle situation	No (%)	Yes (%)
Mental distress	78.9	21.1
Systemic diseases	38.8	61.2
Injuries in the head/neck area/teeth	67.8	32.2
Allergies	79.3	20.7
Smoking	80.7	19.3
Alcohol consumption	52.4	47.6
Drug consumption	97.6	2.4
Existing previous diagnosis	73.1	26.9
Medication prior to first consultation	48.6	51.4

More than half of the study population was referred to the clinic by a private general dentist, 19.2% by their general practitioner, and 9.1% by an ear, nose, and throat (ENT) specialist. Only 5.3% of the patients were self-referred.

### 3.2 Pharmacological therapy prior to orofacial pain consultation

At the initial consultation with the orofacial pain specialist, 51.4% of patients reported the use of pharmacological therapy prior to the first appointment at the Orofacial Pain Unit. Among these patients, 44.9% used NSAIDs prior to diagnosis, 30.8% used non-NSAIDs pain relievers, 9.3% used antidepressants with pain-modulating properties, whereas 18.7% used antidepressants without pain-modulating properties, and 7.5% used magnesium. Only 0.9% of the patients reported the use of topical NSAIDs prior to the consultation at the pain unit. Medication use related to comorbidities was reported by 5.3% of patients, with the specific agents summarized in Table 3.

**Table 3.** Distribution of medications used for comorbidities prior to the orofacial pain consultation.

Medication type	# of patients	%
Z-drug (sleep aid)	1	0.5
Stimulant (ADHD medication)	2	1.0
Analgesic/Sedative/Other	1	0.5
Opioid	1	0.5
Herbal medicine sedative	1	0.5
Herbal medicine	1	0.5
Mood stabilizer/Bipolar disorder treatment	1	0.5
Antibiotic	1	0.5
Lipid-lowering agent (statin)	1	0.5
Beta blocker	1	0.5
Chondroprotective	1	0.5

*ADHD: Attention Deficit Hyperactivity Disorder.*

Age showed to have a significant influence on medication use (*p* = 0.011). Patients between 40 and 60 years of age had the highest use of medication (19.7%), followed by patients between 20 and 40 years of age (17.3%). Presence of psychological distress was significantly associated with higher rates of pharmacological therapy use (*p * < 0.001). Among patients without reported mental distress, 3.8% used pharmacotherapy prior to the consultation in the pain unit; this proportion increased to 16.8% among those with mental distress. Patients experiencing pain in the head region were significantly more likely to have used medication (n = 61) in comparison with those with pain in the face region (n = 40) or in the arm (n = 5) (*p* = 0.023). Patients with higher maximum pain intensity also used more pharmacological therapy prior to their consultation in comparison with patients with lower pain intensity (*p* = 0.005). No significant association was observed between the duration of pain and the use of medication prior to the orofacial pain visit.

### 3.3 Diagnoses distribution before and after orofacial pain consultation

Only 26.4% of the patients presented to the clinic with a previously assigned diagnosis. Of these patients, 31.5% had their diagnosis modified during the consultation, and 61.1% received new diagnoses. Of these cases, the majority (76.5%) were diagnosed with myofascial pain after visiting the Orofacial Pain Unit. Only 7.4% of the pre-existing diagnoses were confirmed (Fig. 1). After receiving a diagnosis at the clinic, the number of patients treated pharmacologically increased significantly to 74.5% (McNemar test, *p * < 0.001).

**Fig. 1.** Changes in previous diagnosis after consultation with the orofacial pain specialist.

After consultation, myofascial orofacial pain was the most common diagnosis assigned by the orofacial pain specialists (50.5%), followed by TMJ pain (22.1%). Diagnoses related to cranial nerve disorders and idiopathic orofacial pain were less common, accounting for 5.8% and 4.3%, respectively (Table 4).

**Table 4.** Main diagnosis received at the orofacial unit.

ICOP Diagnosis	Patients (%)
Orofacial pain or non-painful disorders attributed to disorders of dentoalveolar and anatomically related structures	2.9
Myofascial orofacial pain	50.5
Temporomandibular joint pain and non-painful TMJ disorders	22.1
Orofacial pain attributed to lesion or disease of the cranial nerves	5.8
Orofacial pain resembling presentations of primary headaches	1.9
Idiopathic orofacial pain	4.3
Others (occlusal hypervigilance, obstructive sleep apnoea syndrome, tinnitus)	9.1
No diagnosis	3.4

*TMJ: temporomandibular joint; ICOP: International Classification of Orofacial Pain.*

### 3.4 Therapeutic situation at baseline and therapeutic choices following consultation

The distribution of the pharmacological treatments before and after the diagnosis at the clinic is displayed in Fig. 2. Overall, pharmacotherapy was prescribed to 74.5% of patients. Additionally, 95.7% of the patients received non-pharmacological therapy. The most prescribed type of medication post-diagnosis was NSAIDs with 66.5% (topical: 64%, systemic: 9%), followed by magnesium supplements with 40.0% and antidepressants with pain-modulating properties with 14.8%.

**Fig. 2.** Distribution of medication types pre- and post-first consultation at the pain unit. NSAID: Nonsteroidal Anti-inflammatory Drugs.

The prescription of pharmacological treatment after diagnosis was significantly associated with age (*p* = 0.038), as well as maximum pain intensity (*p* = 0.022) and average pain intensity (*p* = 0.015). No significant association was observed between the duration of pain and the prescription of pharmacological therapy following consultation with the pain specialist.

### 3.5 Changes in pharmacological therapy

Of the total sample of patients, 12.5% had their pharmacological treatment withdrawn, 34.6% received it ex-novo, while 39.9% had their pharmacological treatment maintained and modified if necessary (Fig. 3). Of the patients already using pharmacotherapy (51.4% of the total patients), 19.6% discontinued pharmacological therapy in favour of non-pharmacological therapy (*e.g.*, information therapy, self-observation, and stretching exercises), 4.7% were either referred to other specialists or no follow-up information was available in the charts, and 75.7% continued the pharmacological treatment with the same (8.4%) or different medications (67.3%).

**Fig. 3.** Graphic representation of change in medication for all patients.

Prescription of topical NSAIDs and magnesium supplements showed a significant increase according to McNemar Change Test (*p * < 0.001). Antidepressants with pain modulation effects, in particular amitriptyline, increased significantly (*p* = 0.015), whereas those without pain-modulating properties showed a significant decrease from pre-diagnosis (18.7%) to post-diagnosis (1.3%) (*p * < 0.001). Prescription of systemic NSAIDs and non-NSAID medication significantly decreased after the diagnosis (*p* < 0.001). Steroids also increased from 0 to 6%.

### 3.6 Association between pharmacological therapy changes and diagnoses

Pharmacological therapy occurred significantly more often when the diagnosis was of myofascial orofacial pain, as well as of TMJ pain and non-painful TMJ disorders (*p* = 0.024; *p * < 0.001, respectively).

### 3.7 Effects of therapy

Therapy success could not be measured with changes in the VAS score, as follow up values after treatment were not consistently available. Therefore, the effect of the pharmacological therapy was assessed qualitatively as improvement, worsening, or no effect in 62.6% of patients receiving treatment, due to missing data. Among them, 67.0% improved, in 32.0% the medication showed no effect, and 1.0% reported worsening. The distribution of the medication according to their effect is displayed in Fig. 4.

**Fig. 4.** Pharmacological therapy success across all medications. NSAID: Nonsteroidal Anti-inflammatory Drugs.

Overall, the presence of pharmacological intervention did not show a significant association with therapy success (*p* = 0.304).

## 4. Discussion

The primary aim of this study was to evaluate how often the therapy was changed after a consultation with an orofacial pain specialist at the Orofacial Pain Unit, Center for Dental Medicine, University of Zurich, and thus identify the associated factors that might contribute to pharmacological therapy choices. The statistical results gave an insight on the distribution of pharmacological therapies in the study population, the factors influencing medication use, and the potential issues related to misdiagnosis which can lead to inadequate pharmacological therapy.

Of the 208 patients, 51.4% reported using pharmacotherapy prior to their consultation with an orofacial pain specialist (pre-diagnosis). It is important to note that from this initial group, the majority (67.3%) modified their therapy after meeting the orofacial pain specialist. This highlights a potential risk of improper medication use in orofacial pain patients, especially when medication is taken before a proper diagnosis is formulated. Previous studies showed that up to around 60% of tooth pain and TMD respondents were self-medicating, primarily with analgesics and over-the counter drugs, underlying this tendency in the general population [24, 25]. In our results, the most frequently prescribed medication before consultation with the orofacial pain specialists were systemic NSAIDs (44.9%). This therapeutic decision made by practitioners represents a significant risk, as myofascial pain, which was the most common diagnosis in this study, is usually chronic in nature. Prolonged use of NSAIDs, which are in themselves an acute medication, exposes patients to an increased risk of adverse effects, such as gastrointestinal bleeding. Accordingly, the indication for NSAIDs should be carefully considered. It has also been clearly reported in the literature, that pharmacological treatment strategies differ between TMD of muscular and arthrogenous origin. It is therefore essential to first identify the individual and multifactorial causes underlying each patient’s condition, and only then apply a comprehensive, multimodal treatment plan that includes pharmacological options. In the case of muscular TMD, recent studies support the use of wet needling therapies with botulinum toxin type A (BTX-A), granisetron, platelet-rich plasma (PRP), and muscle relaxants. Conversely, in the case of arthrogenous TMD, the available evidence favours NSAIDs, glucocorticosteroids (in cases of inflammation), hyaluronic acid, and dextrose [26]. For certain inflammatory diseases, such as TMJ osteoarthritis, systemic NSAIDs show high efficacy with a number needed to treat (NNT) of 1.6–3.0 [27].

As for post diagnosis, a significant increase in pharmacological therapy was observed, with topical NSAIDs being the most prescribed medication. A review by Kotowska-Rodziewicz *et al*. [28] underlines the common use of NSAIDs in dentistry and the increasing role of topical applications, showing how they are proving highly effective in managing pain associated with various dental procedures and conditions, including TMJ disorders. Furthermore, it must be noted that non-pharmacological treatment was prescribed in 95% of the cases, alone or in addition to pharmacotherapy. All patients showing signs of mechanical tooth wear due to bruxism received verbal education about bruxism being a possible contributing factor to their pain, written instructions on controlling bruxism when awake, as well as on self-applied physiotherapeutic measures, such as massage, stretching, and warm/cold treatment [29]. Patients reporting nocturnal bruxism were recommended to apply an orthotic device. For patients experiencing mental distress, electromyography-based biofeedback as well as relaxation techniques were advised. There were no specific diagnoses made for mental distress, however patients interested in consulting a psychiatrist received an appropriate referral.

Our results show that several key factors were associated with pharmacological therapy use, including age, psychological distress, pain intensity, as well as location of pain. Patients in the age group between 40 and 60 years showed an increased trend compared with other age groups in pharmacological therapy pre-diagnosis, as reported in other studies [27, 30, 31]. Thus, age, coupled with the absence of an accurate diagnosis, could contribute to the inadequate treatment of orofacial pain patients. Mental distress emerged as a significant factor within the patient population. This is consistent with previous studies reporting a higher prevalence of orofacial pain in patients with psychological comorbidities [32, 33, 34]. Furthermore, the presence of depression and anxiety can even be a predictor for the development orofacial pain [35]. Patients experiencing pain in the head region were more likely to use pharmacotherapy, possibly due to the disability caused by head-related pain, urging patients to seek faster medication. Diagnostic accuracy before the orofacial pain visit was also an issue. Among the 26.4% of the patients that visited the clinic with a previously assigned diagnosis, only 7.4% of them were confirmed by the specialist. This could have potentially played a role in inadequate patient treatments. Research has shown that misdiagnosis in orofacial pain is a significant challenge often leading to the wrong treatment [36]. Furthermore, in 76% of cases where the specialist changed the diagnosis, the new diagnosis was myofascial pain. Hence, myofascial orofacial pain emerges as the most frequently overlooked diagnosis. Furthermore, it represents the clinical entity posing the greatest diagnostic challenge within the spectrum of orofacial pain disorders. The diagnostic limitations are intensified by the questionable reliability of clinical examinations in identifying trigger points, as previously reported, as well as the overall validity of trigger points as the causal mechanism for perceived muscle pain [37, 38]. These considerations underscore the necessity of critically re-evaluating and potentially reconceptualizing this construct in the development of future diagnostic frameworks.

Despite the increase in pharmacotherapy, the new or adjusted regimens did not reflect in significant changes in pain perception, underscoring the multifactorial complexity of orofacial pain. Beyond anatomical challenges, psychological, lifestyle, and social determinants contribute substantially to symptom persistence. These findings reinforce the necessity of an interdisciplinary management strategy and highlight the role of non-pharmacological interventions in optimizing patient outcomes. Furthermore, the presence of comorbidities can have a significant effect on the therapy outcomes.

One major limitation of this study was the lack of long-term follow-up data, which made it difficult to assess the long term effect of the therapy. The cross-sectional design of the study does not allow for drawing conclusions about the causative effects of the observed associated factors. Furthermore, patient selection was conducted over a six-year period of clinical practice, with 40 patients randomly chosen from each year, which may have introduced bias into the results. Finally, investigating non-pharmacological therapy which might be running parallel to the pharmacological one, could prove valuable in future studies, to better elucidate the therapy effects, by eliminating possible confounding factors.

Given the results of the study, the need for broad access to training and further education becomes evident. Since half of the patients were not referred by dentists but by other specialists, it would be important for orofacial pain to be integrated not only into the dental but also into the general medical curriculum. While the formal recognition of orofacial pain as a standalone dental specialty is still developing globally, several countries have established frameworks for its practice within oral medicine or related fields. This trend reflects a growing acknowledgment of the importance of specialized care for patients with chronic orofacial pain conditions. This altogether emphasizes the importance of personalized care in orofacial pain patients, where the expertise of a specialist, coupled with an accurate diagnosis and appropriate treatment, can significantly improve patients’ quality of life and demonstrates the need for patient-centred care.

## 5. Conclusions

This study showed that pharmacological treatment in orofacial pain patients is highly complex, highlighting the importance of an accurate diagnosis and a more personalized treatment in such patients.

● Predictors of pharmacotherapy use before diagnosis include age, psychological distress, and pain location.

● A high rate of misdiagnosis exists among patients with myofascial orofacial pain.

● Topical NSAIDs followed by magnesium supplements were the most prescribed pharmacological therapies after consultation with an orofacial pain specialist.
